# Transphyseal ACL reconstruction and tenodesis in skeletally immature patients demonstrates encouraging clinical scores, without growth disturbance, excessive laxity or re-injury

**DOI:** 10.1016/j.jor.2024.02.028

**Published:** 2024-02-20

**Authors:** Jay R. Ebert, Salar Sobhi, Peter T. Annear

**Affiliations:** a School of Human Sciences (Exercise and Sport Science), University of Western Australia, Crawley, Western, Australia; b HFRC Rehabilitation Clinic, 117 Stirling Highway, Nedlands, Western, Australia; c Perth Orthopaedic & Sports Medicine Research Institute, West Perth, Western, Australia; d Perth Orthopaedic and Sports Medicine Centre, West Perth, Western, Australia

## Abstract

**Purpose:**

Paediatric patients demonstrate high re-rupture rates after anterior cruciate ligament reconstruction (ACLR), with numerous surgical techniques proposed to deal with this challenging cohort. This study investigated the early clinical outcomes, complications, return to sport (RTS) and re-rupture rates up until 2-years post-surgery in paediatric patients presenting with open growth plates undergoing transphyseal ACLR that was combined with an extra-articular tenodesis (LET).

**Methods:**

Between October 2017 and September 2020, 20 skeletally immature patients were consecutively recruited and underwent transphyseal ACLR and LET. Patient reported outcome measures (PROMs), KT-1000 laxity, knee range of motion (ROM), maximal isokinetic knee torque and a 3-hop battery were assessed at 6-, 12- and 24-months. Limb Symmetry Indices (LSIs), RTS rates, complications, re-ruptures and re-operations were reviewed.

**Results:**

All PROMs improved (p < 0.05). No change (p = 0.903) in laxity between limbs was seen, while 18 patients (90%) demonstrated normal (<3 mm) or near normal (3–5 mm) laxity differences. Peak knee flexion ROM improved over time (p = 0.028), while LSIs for knee extensor strength (p < 0.001), the single (p = 0.002) and triple crossover (p = 0.038) hop tests improved. At 24 months, 18 patients (90%) were participating in their pre-injury pivoting sport activities. No complications, growth disturbances, re-injuries or subsequent surgeries were observed.

**Conclusions:**

Transphyseal ACLR combined with LET, undertaken in skeletally immature paediatric patients, demonstrated high scoring PROMs, physical performance and RTS overall, without evidence of growth disturbance or excessive graft laxity. No re-injuries have been observed at this time with ongoing review required in this high-risk cohort.

## 1 Introduction

Anterior cruciate ligament (ACL) rupture and associated reconstruction (ACLR) is common,^1^ though a significant increase in both has been reported in paediatric and adolescent patients.^2^ In the skeletally immature patient, undergoing delayed ACLR or following a non-operative treatment pathway is a concern for further intra-articular injury.^3^, ^4^, ^5^ A trend toward early surgery has been observed in children and adolescents, with non-operative patients experiencing more pathological laxity and unable to return to prior activity levels.^6^ A review and meta-analysis undertaken in young patients reported that delaying ACLR for >12 weeks increased the subsequent risk of concomitant meniscal pathology and irreparable tears, though reconstruction in the early or delayed setting still achieved satisfactory knee stability.^7^

In patients presenting that are yet to mature skeletally, ACLR was traditionally delayed until closer to maturity in the fear of potential growth deformity.^6^^,^^8^, ^9^, ^10^ A range of transphyseal, partial transphyseal and physeal-sparing ACLR techniques may be employed, though regardless of the technique growth disturbances are now less common though failure rates still range from 15 to 25%.^11^ In adults and to mitigate the relatively high rates of graft re-rupture that are also reported, double-bundle (versus single-bundle) ACLR^12^ and concomitant lateral extra-articular tenodesis (LET)^13^ may be employed. Albeit in a cohort 14–25 years of age and not specific to a paediatric cohort, Getgood et al.^13^ demonstrated that adding a LET to ACLR using a hamstrings autograft reduced the risk of persistent instability and graft re-rupture.

This study sought to investigate the early outcomes in a prospectively recruited paediatric cohort with open growth plates undergoing single-bundle transphyseal ACLR, concomitant with LET. It was hypothesized that over a 24-month post-operative period: 1) patient reported outcome measures (PROMs) and physical outcomes would significantly improve, 2) a majority of patients would be actively participating in pre-injury pivoting sports, 3) no excessive post-operative laxity differences would be observed, 4) a low rate of re-rupture (<5%) would be demonstrated, and 5) no growth disturbance issues would be observed.

## 2 Materials and methods

### 2.1 Patients

**Fig. 1:**
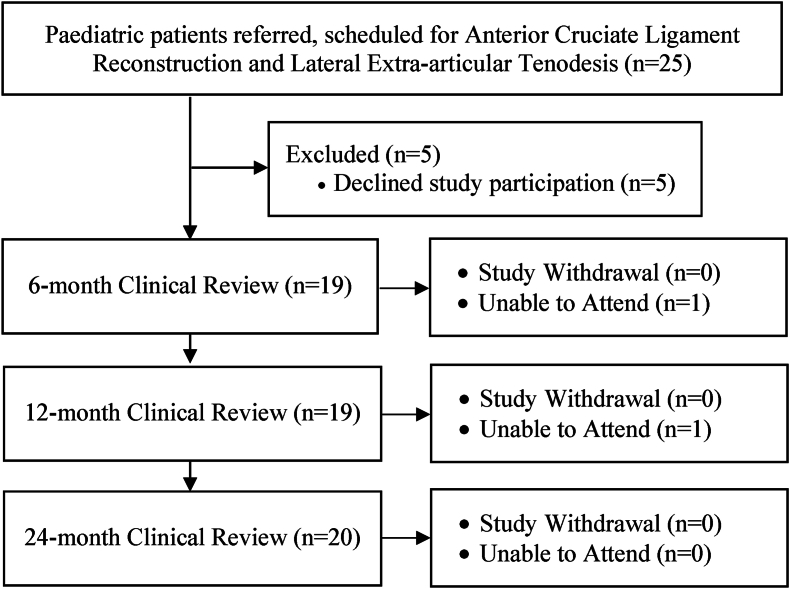
Patient recruitment and assessment over the 24-month period.

Between October 2017 and September 2020, 25 skeletally immature paediatric patients prospectively scheduled for transphyseal ACLR and LET with a single surgeon were referred (Fig. 1). Inclusion criteria included patients scheduled for ACLR concomitant with LET (and meniscal surgery if needed), with ACL rupture confirmed by magnetic resonance imaging (MRI) and specialist examination. Patients were excluded from study participation if they presented with a history of past significant injury on the ipsilateral or contralateral limb, or those with multi-ligamentous injuries. Human Research Ethics Committee (HREC) approval was obtained.

### 2.2 Surgical technique

**Fig. 2:**
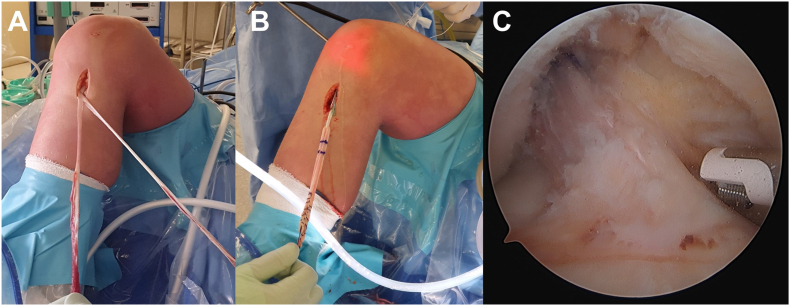
Harvesting of gracilis and semitendinosus hamstrings, noting the long harvested tendons to allow staple fixation on the tibia below the growth plate (A). Following graft preparation of the doubled tendons, passaging of the graft (B) and fixation, the final graft construct can be seen sitting within the stable remnant with no notch impingement (C).

All patients underwent an arthroscopically assisted, single bundle transphyseal ACLR and LET procedure with a high tourniquet. Harvesting of gracilis and semitendinosus hamstrings was undertaken via a 2–3 cm incision, with care taken to optimize tendon length to allow fixation below the tibial growth plate (Fig. 2). The doubled tendons were combined and a 20 mm closed loop button (Endobutton, Smith and Nephew) was employed for femoral fixation. An anteromedial portal was employed to prepare the femoral tunnel. A 10 o'clock position (right knee) 5 mm forward of the intercondylar notch was targeted for femoral tunnel position. A tibial targeting elbow jig was placed to deliver the guide wire centrally into the ACL tibial footprint. Stable ACL remnant and notch synovium was spared, along with fat pad and ligamentum mucosa. Femoral fixation was performed with the closed loop button (Endobutton, Smith and Nephew). The tibial fixation performed was by two 2 prong medium staples (Richards) fixed distally to the tibial physis and medial to the tibia apophysis. Maximal manual tension on the autograft, with the knee in extension was the position for staple fixation. Following graft passage, the tails of the tendons provided length for staple fixation below the tibial physis. The final intra-operative graft position is shown (Fig. 2).

**Fig. 3:**
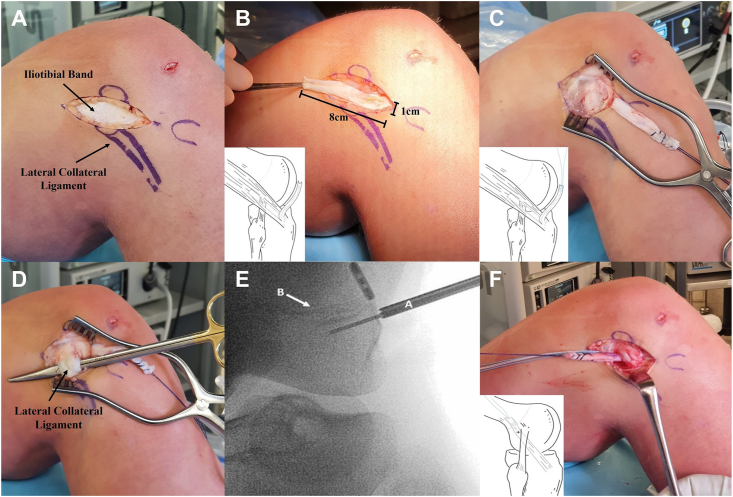
Marking of anatomical landmarks, followed by a 5–8 cm longitudinal incision being made on the inferior aspect of the lateral epicondyle in line with Gerdy's tubercle (A). A 1 cm by 8 cm ITB strip was harvested proximally while maintaining the distal attachment to Gerdy's tubercle (B), with the proximal end of the ITB graft whip stitched with 1–0 Vicryl (C). Blunt dissection with scissors creates a passage deep to the proximal LCL for graft passage (D). The distally directed awl is positioned with suspensory fixation for the hamstring ACLR graft above the physis (E), with the ITB graft passaged deep to the proximal LCL (F).

Following ACLR, the LET procedure was undertaken in 90° of knee flexion. Gerdy's tubercle, the lateral epicondyle and the fibular collateral ligament were marked, and a 5–8 cm incision was made inferior of the lateral epicondyle in line with Gerdy's tubercle (Fig. 3). A 1 cm × 8 cm ITB strip was harvested proximally while maintaining the distal attachment to Gerdy's tubercle (Fig. 3). Subsequently, the proximal end of the ITB graft was whip stitched with 1–0 Vicryl (Fig. 3). The proximal attachment of the fibular collateral ligament was then dissected to define the LCL attachment, while blunt dissection with scissors created a passage deep to the proximal LCL to allow graft passage (Fig. 3). The bone anchor awl (Twin Fix-Smith, Smith and Nephew) was placed proximal to the edge of the LCL and 5–10 mm posterior to the LCL femoral attachment. The awl was directed distally to avoid the distal femoral physis (Fig. 3) and the correct position was confirmed by intra-operative image intensifier. A bone anchor with sutures was placed (Twin Fix-Smith, Smith and Nephew) in the awl hole. The ITB graft was then passaged deep to the proximal LCL (Fig. 3) and fixed to the bone anchor at 30° of knee flexion. The knee was ranged to confirm full extension prior to final suture tension/fixation, with the excess graft excised.

### 2.3 Rehabilitation

Patients were managed post-operatively with early bracing and touch weight-bearing for 3–6 weeks. Early circulation and knee motion exercises commenced immediately and cycling, proprioceptive, strengthening and closed-chain exercises from 5 to 6 weeks. End-range open-chain exercises were permitted from 4 months, along with running and higher-level rehabilitation activities. Exercise progression was based on several variables including time from surgery, concomitant meniscal surgery, proficiency of rehabilitation activities, pain and effusion, quadriceps control and knee ROM. RTS was generally permitted from 12 months, though following consultation between the patient (and family), surgeon and therapist and considering factors such as pain and effusion, full or near-full restoration of active knee ROM, and the restoration of a sound level of strength and physical capacity.

### 2.4 Subjective assessment

The Paediatric International Knee Documentation Committee (pedi-IKDC) form^14^ and the Knee Outcome Survey Activities of Daily Living (KOS-ADL) Scale^15^ were assessed at 6-, 12- and 24-months. A review of sports participation was undertaken at all time-points.

### 2.5 Objective assessment

The senior author undertook a formal clinical examination in all patients at 4 months. A knee arthrometer (KT-1000, MEDmetric Corp., San Diego, CA, USA) was employed to assess tibial translation (mm) during a maximal manual test (MMT) on both limbs at 6-, 12- and 24-months. Active knee flexion and extension ROM was assessed. Patients underwent the single (SHD, m), triple (THD, m) and triple crossover (TCHD, m) hop tests for distance,^16^ along with the assessment of maximal isokinetic knee extensor and flexor torque (Isosport, Gepps Cross, South Australia).

### 2.6 Complications

Surgical complications, re-ruptures and re-operations were presented. At 6-, 12- and 24-months, visual assessment of lower limb angular deformity in standing assessed side-to-side differences, while limb length was assessed in supine using a tape measure from the anterior superior iliac spine to the both the medial and lateral malleolus. While the decision to refer for more accurate radiological limb length and alignment examination was based on obvious side-to-side limb length differences ≥1 cm as reported by Collins et al.,^17^ this was not encountered.

### 2.7 Data and statistical analysis

The required sample size was calculated based on the Limb Symmetry Index (LSI) of the SHD at 6-months. An LSI <90% is generally considered unsatisfactory^18^, ^19^, ^20^; therefore, anticipating the LSI to be 100% normally and accepting side-to-side equivalence if the LSI is >90%, using these values (i.e. 105 non-inferiority margin) and a SD of 12% (attained via pre-existing data for the SHD in a paediatric cohort), 18 patients were required at alpha 0.05 with 90% power to test the primary non-inferiority hypothesis.

Changes in PROMs and physical measures were assessed over time via analysis of variance (ANOVA). Limb Symmetry Indices (LSIs) were calculated for the physical performance measures. KT-1000 measurements were categorized based on differences between limbs as normal (<3 mm), nearly normal (3–5 mm), abnormal (6–10 mm) and severely abnormal (>10 mm).^21^ ANOVA was employed to assess laxity changes over the post-operative period. SPSS (SPSS, Version 27.0, SPSS Inc., USA) was employed for statistical procedures, with statistical significance at *p* < 0.05.

## 3 Results

Characteristics of the recruited cohort are demonstrated (Table 1), while Fig. 1 outlines the recruitment and assessment pathway of study patients.

**Table 1:** Patient demographics and injury/surgery characteristics for the cohort recruited.

Variable	Measure	n = 20
Age (y)	Mean (SD)	13.1 (1.8)
	Range	8–15
Body Mass Index	Mean (SD)	22.0 (4.0)
	Range	16.2–31.8
Injury to Surgery Time (weeks)	Mean (SD)	24.5 (46.0)
	Range	4–156
Gender (males)	n (%)	14 (70.0)
Injury Mechanism (non-contact)	n (%)	16 (80.0)
Adjunct Surgery	n (%)	8 (40.0)
Meniscectomy	n (%)	2 (10.0)
Meniscus Repair	n (%)	6 (30.0)

### 3.1 Subjective outcomes and return to sport

All PROMs improved over the period (p < 0.0001) (Table 2). At 24-months, 18 patients (90%) were actively participating in their pre-injury pivoting sport activities, including Australian Rules Football (n = 6), basketball (n = 4), rugby (n = 2), martial arts (n = 2), soccer (n = 1), netball (n = 1), gymnastics (n = 1) and athletics (n = 1). One further patient was undertaking competitive BMX, while one patient was participating in full AFL training activities yet to play a game.

**Table 2:** Subjective scores and knee range of motion (degrees) for the operated and non-operated limbs, throughout the post-operative timeline. Shown are means (SD), with p-values representing the change over time.

Time-point	pedi-IKDC (0–100)	KOS-ADL (0–80)	Knee Flexion (deg)		Knee Extension (deg)	
			Operated	Non-operated	Operated	Non-operated
6 months	81.4 (7.7)	74.2 (3.6)	141.2 (7.7)	150.2 (6.0)	−1.5 (2.0)	−2.5 (2.8)
12 months	93.8 (7.2)	78.2 (2.3)	143.6 (6.2)	150.3 (6.5)	−1.5 (2.1)	−2.3 (2.3)
24 months	97.6 (2.9)	79.0 (1.5)	146.8 (4.8)	150.2 (5.5)	−2.1 (2.2)	−2.4 (2.3)
p-value	<0.0001	<0.0001	0.028	0.996	0.619	0.966

Pedi-IKDC = Paediatric International Knee Documentation Committee Form (pedi-IKDC); KOS-ADL = Knee Outcome Survey Activities of Daily Living.

### 3.2 Objective results

At 4 months all patients had a Grade 0–1 pivot shift examination. No change (p = 0.903) in laxity differences between limbs was seen over time, while 18 patients (90%) demonstrated normal (<3 mm) or near normal (3–5 mm) KT-1000 side-to-side differences (Table 3). The LSIs for knee extensor strength (p < 0.001), the SHD (p = 0.002) and the TCHD (p = 0.038) improved over time (Table 4).

**Table 3:** KT-1000 side-to-side difference (mm) scores at 6-, 12- and 24-months. The number (and percentage) of knees graded normal (<3 mm), nearly normal (3–5 mm) and abnormal (6–10 mm) is shown. No knees demonstrated severely abnormal (>10 mm) scores.

Variable	Measure	6 months (n = 19)	12 months (n = 19)	24 months (n = 20)	p value
KT-1000, side-to-side difference (mm)	Mean (SD), range	2.1 (1.9), 0-7	2.2 (2.0), 0-7	2.4 (1.9), 0-7	0.903
Normal (<3 mm)	n (%)	16 (84.2)	16 (84.2)	15 (75.0)	N/A
Nearly normal (3–5 mm)	n (%)	1 (5.3)	1 (5.3)	3 (15.0)	N/A
Abnormal (6–10 mm)	n (%)	2 (10.5)	2 (10.5)	2 (10.0)	N/A

**Table 4:** Mean limb symmetry indices (LSIs) for maximal knee flexor and extensor strength, as well as the single (SHD), triple (THD) and triple crossover (TCHD) hop tests at 6-, 12- and 24-months. Means (SD) are shown, with p-values representing the change over time.

Time-point	Knee Extensor Torque LSI	Knee Flexor Torque LSI	SHD LSI	THD LSI	TCHD LSI
6 months	82.5 (8.9)	90.0 (11.8)	91.5 (6.6)	93.2 (4.5)	89.9 (7.3)
12 months	91.6 (7.7)	95.4 (8.7)	97.0 (7.0)	96.9 (5.6)	94.5 (6.3)
24 months	95.7 (6.3)	94.9 (4.7)	98.7 (4.5)	96.9 (4.3)	96.1 (6.7)
p value	<0.0001	0.121	0.002	0.071	0.038

### 3.3 Complications

No surgical or post-operative complications or adverse events were observed. No patient demonstrated a side-to-side limb length difference ≥1 cm which reflected our clinical criteria for post-operative radiological review of limb growth disturbance. No patient required subsequent surgery, nor were any ipsilateral graft re-ruptures or contralateral tears observed.

## 4 Discussion

The current study demonstrated that transphyseal ACLR combined with LET, undertaken in skeletally immature patients, resulted in high scoring early PROMs and physical performance recovery, without evidence of growth disturbance or excessive side-to-side graft laxity. No subsequent surgeries or re-injuries were observed at 24 months despite the high RTS rate.

PROMs and the majority of objective measures significantly improved over the post-operative timeline, largely supporting the first hypothesis. While a scoping review^22^ reported limited evidence at this time for objective and RTS testing in children and adolescents <16 years, it was encouraging that the current cohort appeared to have recovered a satisfactory level of physical strength/function. Existing research in an adult population has shown an increased re-rupture risk if patients are unable to meet ≥90% LSIs during physical performance measures,^23^^,^^24^ including those employed in the current study.

All patients had returned to pivoting activities within 24 months, with 90% of patients having returned and actively participating in their pre-injury pivoting competitive sports. This was in support of the second hypothesis. It has been reported in previous research that only 65% of patients overall return to a sporting level similar to their pre-injury state, with 55% returning to competitive sport.^25^ A recent review reported that in children and adolescents after ACLR, 79% return to a pre-injury level of sport and 81% to a competitive level of sport.^26^

The higher rate of RTS may also be associated with a greater prevalence of re-injury, with a meta-analysis in 2019 reporting an 8.7% re-rupture rate after paediatric ACLR,^27^ with a systematic review and meta-analysis published in 2018 reporting a 13% ipsilateral re-tear rate and a 14% contralateral 10.13039/100006307ACL tear rate when pooling children and adolescents (6–19 years).^26^ The high RTS rate in the current study by 24 months was not associated with excessive graft laxity or an elevated re-rupture risk at this time, supporting the third and fourth hypotheses. The association between excessive knee laxity and worse knee-related quality of life, reduced sports function and an increased rate of re-operation has been previously reported.^28^ Nonetheless, the risk of graft re-rupture may extend well after the patient's RTS so ongoing review of this higher-risk cohort is required.

No complications were observed. While growth disturbances are now less common but can occur when employing physeal-sparing, partial transphyseal and transphyseal ACLR techniques,^11^ a meta-analysis investigating complications after paediatric ACLR reported a low 4.4% total growth disturbance rate.^27^ The authors suggested that proper surgical technique was likely more important than the actual technique employed.^27^ No patient in the current cohort demonstrated a side-to-side limb length difference ≥1 cm,^17^ which reflected our clinical criteria for radiological review of limb growth disturbance.

A number of limitations are acknowledged. Firstly, it was undertaken in a relatively small sample, with 24-month follow-up and there was no comparative group. Secondly, we acknowledge that formal post-operative radiological review to assess growth disturbance was not undertaken also as per the senior author's standard clinical pathway, and only deemed appropriate if clinical review detected side-to-side limb differences ≥1 cm as per reported criteria.^17^ Thirdly, while aforementioned standardized guidance was provided to patients and their families regarding rehabilitation and RTS, these were community level ACLR paediatric patients undergoing rehabilitation with different therapists and within varied rehabilitation environments. Finally, while it has been reported that objective review and clearance for RTS in paediatric patients following ACLR should be based on a test battery including strength tests, movement quality and paediatric PROMs, limited evidence exists at this time for objective and RTS testing in children and adolescents under 16 years of age.^22^

## 5 Conclusions

This study demonstrated that transphyseal ACLR combined with LET, undertaken in skeletally immature paediatric patients, demonstrated high scoring PROMs and physical performance recovery, without evidence of growth disturbance or excessive side-to-side graft laxity. No re-injuries were observed at 24 months despite the high RTS rate. Given re-injury risk may extend well after the patient's RTS, especially in this higher-risk young ACLR cohort, longer-term follow-up is required.

## Credit author statement

In accordance with the instructions to authors for the journal, author contributions toward this study andmanuscript are as follows.

• Jay Ebert, PhD (Contribution: study design and oversight/organization, performed clinicalmeasurements, statistical analysis, preparation of draft manuscript, review and approval of finalmanuscript)
• Salar Sobhi, MD (Contribution: study design, statistical analysis, preparation of draftmanuscript, review and approval of final manuscript)
• Peter Annear, MD (Contribution: study design and senior oversight, performed clinicalmeasurements, review and approval of final manuscript)

I can confirm that all authors satisfy the below nominated criteria for authorship.

• Substantial contributions to the conception or design of the work; or the acquisition, analysis, or interpretation of data for the work; and
• Drafting the work or revising it critically for important intellectual content; and
• Final approval of the version to be published; and
• Agreement to be accountable for all aspects of the work in ensuring that questions related tothe accuracy or integrity of any part of the work are appropriately investigated and resolved.

## Author contributions

The following authors have conceived and designed the study (JE; SS; PA), supervised the conduct of the study (JE; SS; PA), analyzed the data (JE; SS), wrote the initial drafts (JE; SS), critically revised the manuscript (JE; SS; PA) and ensure the accuracy of the data and analysis (JE; SS; PA). I confirm that all authors have seen and agree with the contents of the manuscript and agree that work has not been submitted or published elsewhere in whole or part.

## Ethical statement

All procedures for the study, including parental/patient consent, were undertaken as per the institutional approval obtained for this study from the Hollywood Private Hospital Human Research Ethics Committee (HPH382).

## Funding statement

No funding was provided for this study.

## Guardian/parental/patient consent

All procedures for the study, including parental/patient consent, were undertaken as per the institutional approval obtained for this study from the Hollywood Private Hospital Human Research Ethics Committee (HPH382).

## Declaration of competing interest

We wish to confirm that there are no known conflicts of interest associated with this publication, though independent funding in the form of a research grant was provided by ConMed Corporation to assist this research.

We confirm that the manuscript has been read and approved by all named authors (as stated in our Cover Letter) and that there are no other persons who satisfied the criteria for authorship but are not listed.

We further confirm that the order of authors listed in the manuscript has been approved by all of us.

We confirm that we have given due consideration to the protection of intellectual property associated with this work and that there are no impediments to publication, including the timing of publication, with respect to intellectual property. In so doing we confirm that we have followed the regulations of our institutions concerning intellectual property. We further confirm that any aspect of the work covered in this manuscript that has involved either experimental animals or human patients has been conducted with the ethical approval of all relevant bodies and that such approvals are acknowledged within the manuscript.

We understand that the Corresponding Author is the sole contact for the Editorial process (including Editorial Manager and direct communications with the office). He is responsible for communicating with the other authors about progress, submissions of revisions and final approval of proofs. We confirm that we have provided a current, correct email address which is accessible by the Corresponding Author and which has been configured to accept email from jay.ebert@uwa.edu.au.mailto:jay.ebert@uwa.edu.au
